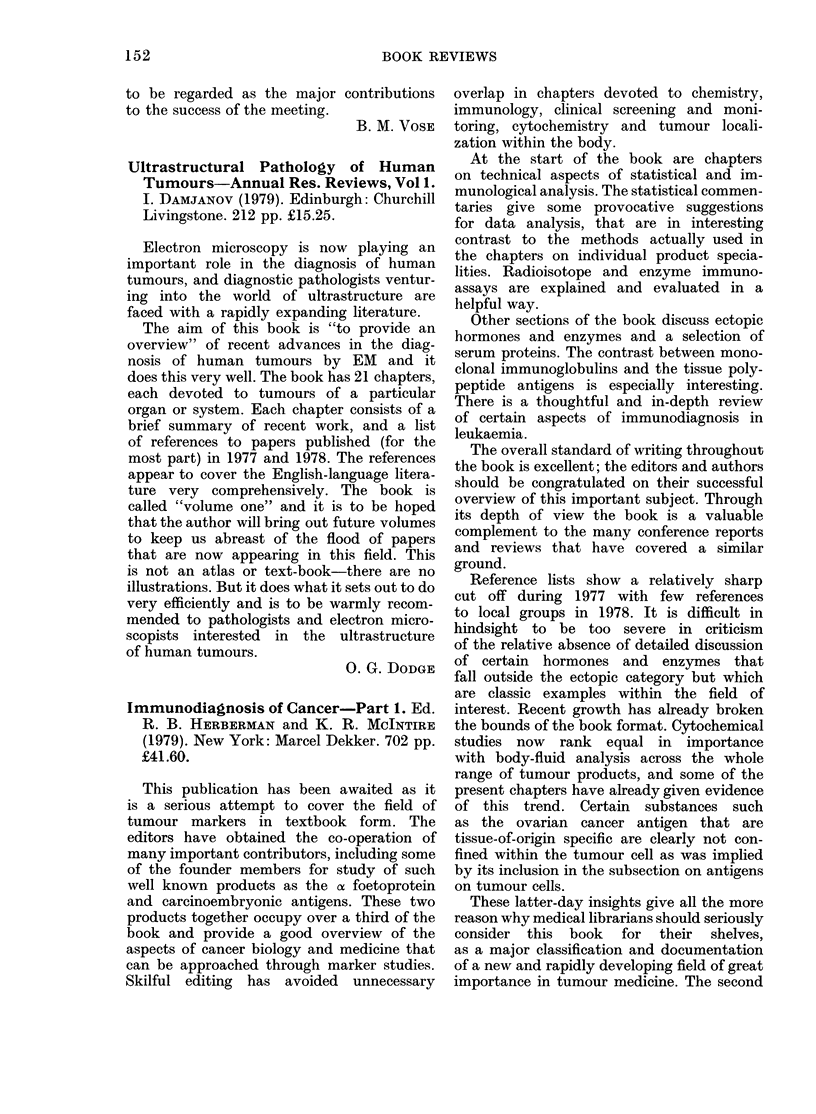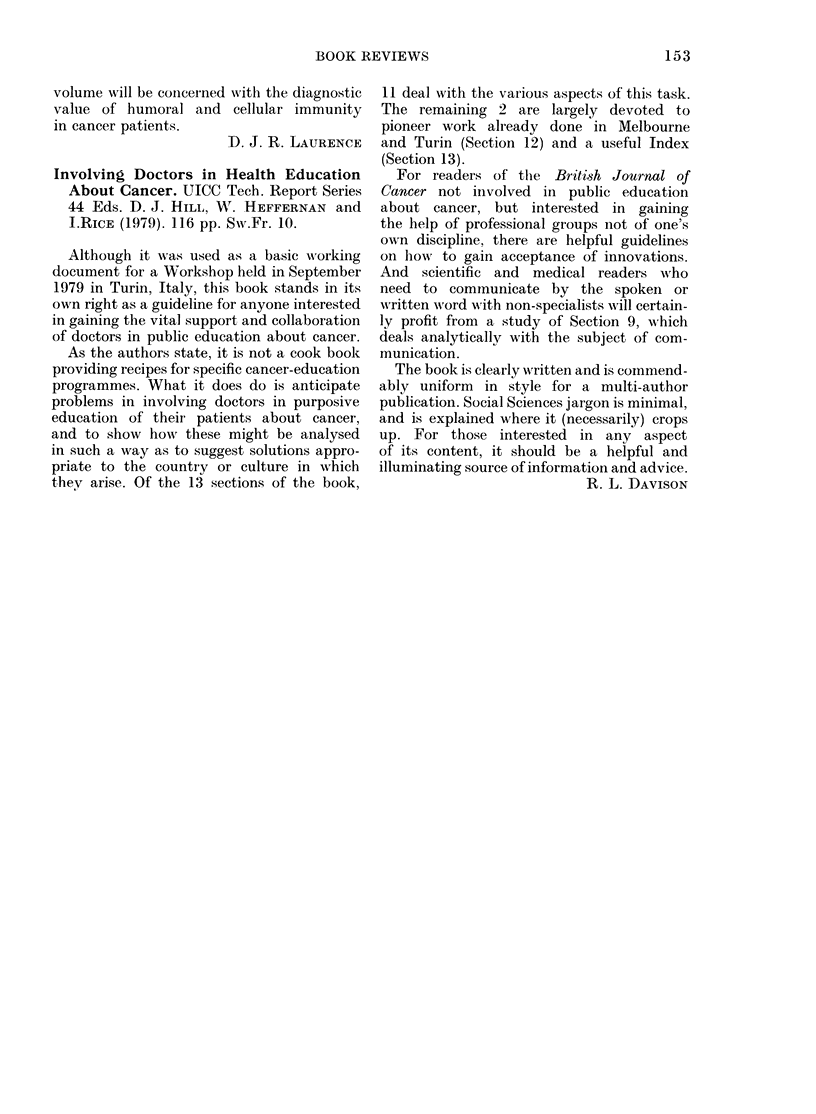# Immunodiagnosis of Cancer—Part 1

**Published:** 1980-01

**Authors:** D. J. R. Laurence


**Immunodiagnosis of Cancer—Part 1.** Ed. R. B. HERBERMAN and K. R. MCINTIRE (1979). New York: Marcel Dekker. 702 pp. £41.60.

This publication has been awaited as it is a serious attempt to cover the field of tumour markers in textbook form. The editors have obtained the co-operation of many important contributors, including some of the founder members for study of such well known products as the $\alpha$ foetoprotein and carcinoembryonic antigens. These two products together occupy over a third of the book and provide a good overview of the aspects of cancer biology and medicine that can be approached through marker studies. Skilful editing has avoided unnecessary overlap in chapters devoted to chemistry, immunology, clinical screening and monitoring, cytochemistry and tumour localization within the body.

At the start of the book are chapters on technical aspects of statistical and immunological analysis. The statistical commentaries give some provocative suggestions for data analysis, that are in interesting contrast to the methods actually used in the chapters on individual product specialities. Radioisotope and enzyme immunoassays are explained and evaluated in a helpful way.

Other sections of the book discuss ectopic hormones and enzymes and a selection of serum proteins. The contrast between monoclonal immunoglobulins and the tissue polypeptide antigens is especially interesting. There is a thoughtful and in-depth review of certain aspects of immunodiagnosis in leukaemia.

The overall standard of writing throughout the book is excellent; the editors and authors should be congratulated on their successful overview of this important subject. Through its depth of view the book is a valuable complement to the many conference reports and reviews that have covered a similar ground.

Reference lists show a relatively sharp cut off during 1977 with few references to local groups in 1978. It is difficult in hindsight to be too severe in criticism of the relative absence of detailed discussion of certain hormones and enzymes that fall outside the ectopic category but which are classic examples within the field of interest. Recent growth has already broken the bounds of the book format. Cytochemical studies now rank equal in importance with body-fluid analysis across the whole range of tumour products, and some of the present chapters have already given evidence of this trend. Certain substances such as the ovarian cancer antigen that are tissue-of-origin specific are clearly not confined within the tumour cell as was implied by its inclusion in the subsection on antigens on tumour cells.

These latter-day insights give all the more reason why medical librarians should seriously consider this book for their shelves, as a major classification and documentation of a new and rapidly developing field of great importance in tumour medicine. The second volume will be concerned with the diagnostic value of humoral and cellular immunity in cancer patients.

D. J. R. LAURENCE